# Supplementary figures and images for: Estimating force of infection from serologic surveys with imperfect tests
Source: PLoS One. 2021 Mar 4;16(3):e0247255. doi: 10.1371/journal.pone.0247255 (PMC7932155; doi:10.1371/journal.pone.0247255)

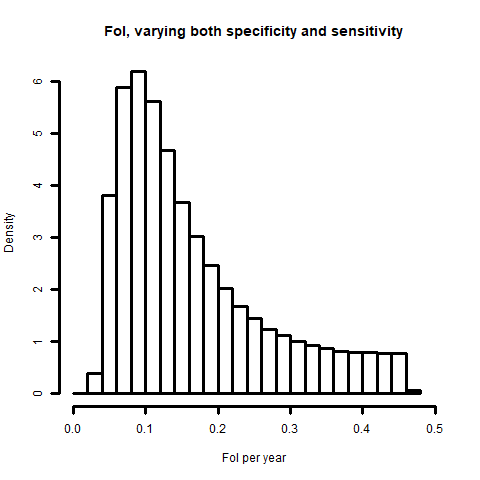

Supplement: S1 Fig — (TIF) [file pone.0247255.s006.tif]
